# SERS-active silver colloids prepared by reduction of silver nitrate with short-chain polyethylene glycol

**DOI:** 10.1186/1556-276X-8-47

**Published:** 2013-01-23

**Authors:** Rares Stiufiuc, Cristian Iacovita, Constantin M Lucaciu, Gabriela Stiufiuc, Alina G Dutu, Cristiana Braescu, Nicolae Leopold

**Affiliations:** 1 Pharmaceutical-Biophysics Department, University of Medicine and Pharmacy ‘Iuliu Hatieganu’, Pasteur 6, Cluj-Napoca, 400349, Romania; 2 Regional Institute of Gastroenterology-Hepatology ‘Octavian Fodor’, Constanta 5, Cluj-Napoca, 400158, Romania; 3 Faculty of Physics, ‘Babes-Bolyai’ University, Kogalniceanu 1, Cluj-Napoca, 400084, Romania

**Keywords:** Silver colloids, Polyethylene glycol, SERS, TEM, UV–vis

## Abstract

We report a fast, one-step, facile, and green preparation method that yields very stable and biocompatible silver colloids that are highly active as surface-enhanced Raman spectroscopy (SERS) platforms that has a possible application in biomedicine. Reduction of silver nitrate has been carried out using polyethylene glycol (PEG) which acts as both reducing agent and stabilizer. It turned out that the -OH groups provided by the addition of NaOH represent a key element in the successful synthesis of PEG-coated silver nanoparticles (AgNPs). The as-obtained silver colloids have been characterized by UV-visible spectroscopy, transmission electron spectroscopy, and SERS using 532- and 633-nm laser lines on a dispersive Raman spectrometer. Several analytes as methylene blue, *p*-aminothiophenol, amoxicillin, and Cu(PAR)_2_ were used to prove SERS enhancement of the obtained silver colloid. It has been found that the PEGylated AgNPs provide SERS signals comparable to those achieved using classical hydroxylamine and citrate-reduced silver colloids, thus demonstrating the ability of this new method to prepare biocompatible silver colloids.

## Background

Since its discovery in 1974, surface-enhanced Raman spectroscopy (SERS) has become a widely used analytical technique offering many advantages over other techniques such as FT-IR spectroscopy, UV-visible-near infrared (UV–vis-NIR) absorption, X-ray photoelectron spectroscopy, mass spectrometry, etc. In the last few years, SERS became very popular in life science applications due to a great amount of information extracted from complex biological environments such as tissues, cell cultures, and biological fluids [1-3]. Although numerous surfaces have been successfully tested as SERS-active substrates (Ag, Au, Cu, Na, Li, Pd, Pt) [4], the best results for biomedical applications have been observed in the case of silver and gold nanoparticles [5]. Compared with gold, silver offers two major advantages: the SERS enhancement factor is 10 to 100 times higher, and it can be excited from the UV to the infrared (IR) region, while gold is restricted to the IR due to the damping induced by interband transitions [6] which have to be taken into account at the nanoscale.

The preparation of silver nanoparticles (AgNPs) is commonly done by reducing the silver ions of a precursor in a solution, usually aqueous media, and preventing particle growth by utilizing stabilizing agents such as surfactants and polymers. In this line, efficient methods of AgNP synthesis have been developed, i.e., the chemical reduction of silver salt solution by a reducing agent such as citrate, NaBH_4_, hydrazine, and hydroxylamine hydrochloride [7-9]. Moreover, given the enormous potential of these nanoparticles in biomedical applications envisaged in the last few years, a more biological approach has been developed for AgNP synthesis by functionalizing them with various biomedical and pharmaceutical substances able to enhance their absorption into malign cells. For proper application in *in vivo* experiments, these novel nanoparticles must overcome several challenging requirements such as biocompatibility, stability in physiological solutions, non-toxicity, and ability to traverse biological barriers. A general strategy employed by many research groups in fulfilling these requirements is based on coating the nanoparticles with different classes of biopolymers. Since polyethylene glycol (PEG) is one of the most versatile biopolymer, environmentally benign and already used in the pharmaceutical and biomedical industries, much of the research interest has been focused on developing new methods of PEGylation. The successful attachment of PEG molecules onto the nanoparticle surface has already been done by adding SH-modified PEG molecules on previously synthesized AgNPs [10] or using PEG as both reducing and stabilizing agents without [11-13] or within aqueous media [14,15]. Although the already reported methods are successful, they have two major drawbacks: the time required for the complete formation of PEG-functionalized AgNPs can reach several hours, and the methodology is quite complex in most of the cases.

In this paper, we report a simple, green, effective, and extremely fast method in preparing stable, highly SERS-active, and biocompatible silver colloids by the reduction of silver nitrate with PEG 200 at alkaline pH in aqueous media. The addition of sodium hydroxide shifts the solution pH towards the alkaline environment, thus reducing the reaction time from several hours to a few seconds. Sequential studies certified that the use of unmodified PEG molecules as reducing agent allows the successful formation of AgNPs. The key element of our method is in the presence of additional -OH groups generated in the solution by sodium hydroxide, enhancing the speed of chemical reduction of silver ions. Astonishing is the fact that Ag^+^ can be steadily reduced to Ag^0^ in such mild conditions, and remarkable is the fact that direct and cleaner AgNPs have been synthesized in a few seconds without using any mediators in the process. The as-produced silver colloids have been characterized by UV–vis spectrometry, transmission electron microscopy (TEM), and SERS. The SERS activity of silver colloids was tested using various analytes and was compared with those given by both citrate- and hydroxylamine-reduced silver colloids.

## Methods

Silver nitrate (0.017 g), PEG 200 (0.680 ml), sodium hydroxide (1.1 ml, 0.1%), amoxicillin, sodium citrate dehydrate, and hydroxylamine hydrochloride were of analytical reagent grade. Double-distilled water (100 ml) was used as solvent. 4-(2-Pyridylazo)resorcinol (PAR) complexes with Cu(II) were prepared by mixing solutions of Cu(II) sulfate pentahydrate and PAR at 1:1 molar ratios, resulting in Cu(PAR)_2_ complexes.

UV–vis spectra were recorded on a UV–vis-NIR diode array spectrometer (ABL&E Jasco Romania S.R.L, Cluj-Napoca, Romania) using standard quartz cells at room temperature. TEM images were taken on an analytical transmission electron microscope ZEISS LIBRA 200 FE (Carl Zeiss Microscopy GmbH, Jena, Germany) operating at 200 kV. A drop of aqueous suspension containing PEG-coated AgNPs was deposited on carbon-coated Cu grid. Excess water was remove by filter paper, and then the sample was left to dry under ambient air. SERS spectra were recorded using Advantage 532 and Advantage 200A Raman spectrometers (DeltaNu, Laramie, WY, USA) equipped with a double frequency NdYAG laser emitting at 532 nm (5-mW laser power) and a HeNe laser emitting at 632.8 nm (4-mW laser power), respectively. The spectral resolution of the two spectrometers was 10 cm^−1^. All the SERS spectra were recorded in 1-ml glass vials filled with 475 μl of silver colloid and 25 μl of analyte. Accumulation times between 0.1 to 40 s were used, the final spectrum being the average of previous four recordings.

## Results and discussion

### PEG-reduced silver colloids

PEG 200 (600 μl) and NaOH 1% (500 μl) were added to 90 ml of water in an Erlenmeyer glass and heated to boil on a magnetic stirrer with heating option. A 10-ml aqueous solution containing 0.017 g AgNO_3_ was then added rapidly or dropwise using a syringe, under vigorous stirring. The formation of AgNPs started immediately, as proven by a significant color shift of the solution towards a light yellow, thus suggesting that the chemical reaction took place and that the seeds are available in the solution. The UV–vis spectra recorded on a sample taken straight after the color shift exhibit a peak located close to 400 nm, thus providing the existence of PEG-reduced AgNPs in the solution. The pH right after preparation was 8, but in time, a slight lowering of the pH was observed. Several days after preparation (at the moment when the SERS spectra were recorded), the pH of the PEG-reduced colloid was 7.5. Several colloids have been prepared using different PEG 200 volumes between 340 and 680 μl. All colloids were found to be SERS active. A volume of 600 μl PEG 200 was found to be an optimum in terms of time stability and SERS enhancement. The calculated molar concentration of the PEG-coated AgNPs was 4 × 10^−9^ M [16].

### Hydroxylamine-reduced silver colloids

Briefly, 0.017 g of silver nitrate was solved in 90 ml of water. In a separate recipient, 0.017 g of hydroxylamine hydrochloride was solved in 10 ml of water, followed by the addition of 1.150 ml 1% sodium hydroxide solution. The hydroxylamine/sodium hydroxide solution was then added rapidly to the silver nitrate solution under vigorous stirring. After a few seconds, a gray-brown colloidal solution was produced, which was further stirred for 10 min. The pH value of the silver colloid, measured immediately after preparation, was found to be 8. Also, a slight lowering of the pH was observed, i.e., at measuring time, the pH was 7.5 [9].

### Citrate-reduced silver colloids

Lee-Meisel method was employed in order to prepare citrate-reduced silver colloids [7]. Briefly, 90 ml of water containing 0.017 mg AgNO_3_ was heated to boil. Afterwards, 10 ml of aqueous solution containing 0.020 g sodium citrate dihydrate was added dropwise under vigorous stirring. At the moment of performing measurements, the pH of the colloid was 7.

### UV–vis spectroscopy and TEM

**Figure 1:**
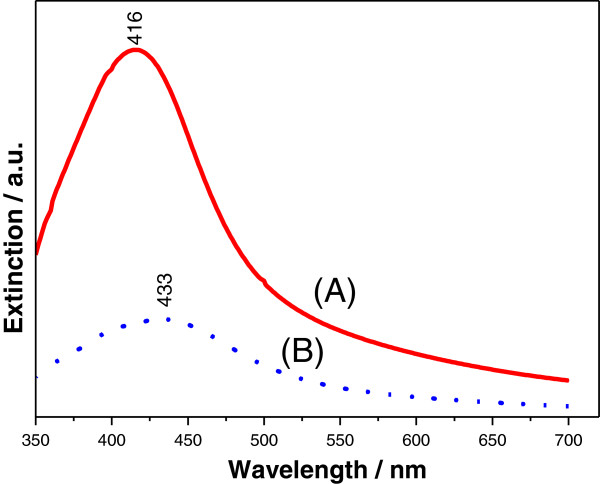
**UV–vis spectroscopy.** UV–vis spectra of PEG-coated AgNPs obtained by either rapid (curve **A**) or dropwise (curve **B**) addition of AgNO_3_ to an aqueous PEG solution. The single peak in both spectra indicates the successful formation of spherical nanoparticles.

In order to characterize the morphology of the produced colloids, UV–vis spectroscopy and TEM were employed. Information on the average particle size can be obtained from the absorption maximum of the measured UV–vis spectrum of the colloidal solution, whereas its full width at half maximum (FWHM) can be used to estimate particle dispersion. It was found that colloids with different particle size and dispersion could be obtained reproducibly by changing the addition time of AgNO_3_ to the aqueous PEG solution. The UV–vis spectrum of the AgNPs synthesized by rapid addition of AgNO_3_ to the aqueous PEG solution exhibits a narrow absorption peak at 416 nm, with an FWHM of approximately 80 nm due to plasmon resonance (Figure 1 curve A), indicating a narrow size and shape distribution immediately post synthesis [17]. The existence of a single surface plasmon resonance peak in the UV–vis spectrum indicates the successful synthesis of the spherical PEG-coated AgNPs. It is worth mentioning that the UV–vis spectrum of the PEG-coated AgNP colloidal solution remained unchanged over several months, indicating that the PEG-coated AgNPs become very stable in time. The PEG molecules that are bound to the silver nanoparticles increase the steric distance between nanoparticles and their hydrophilicity by forming hydrogen bonds with the solvent, thus preventing their aggregation [18]. If the AgNO_3_ is added dropwise to the aqueous PEG solution, the maximum of the absorption band is significantly shifted to 433 nm while the resonance becomes broad (Figure 1 curve B). The redshift reflects the production of the larger-sized AgNPs. The FWHM extends over 100 nm indicating polydisperse silver nanoparticles.

**Figure 2:**
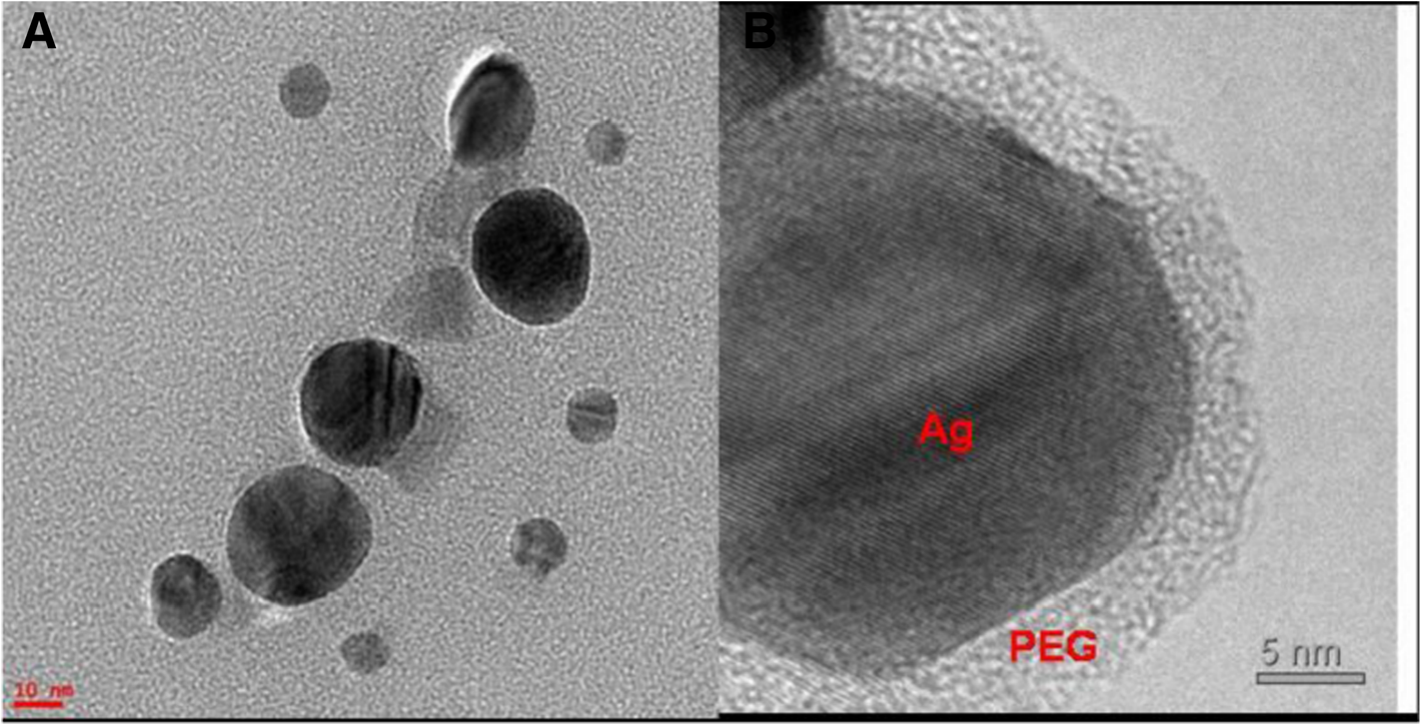
**TEM characterization.** (**A**) TEM images of PEG-reduced AgNPs obtained by rapidly adding AgNO_3_ to the aqueous PEG solution. (**B**) Atomic-scale resolution TEM image of one PEG-reduced AgNP exhibiting the 5-nm PEG layer around the silver core. Spherical PEG-coated AgNPs of narrow size distribution are visible.

Various biomedical applications require biocompatible AgNPs with a narrow size distribution, which, in our case, is achieved by rapid addition of AgNO_3_ to the aqueous PEG solution. Indeed, TEM characterization of the colloidal solution prepared by rapidly adding AgNO_3_ to aqueous PEG solution exhibit PEG-coated AgNPs with diameters between 10 and 30 nm (Figure 2A), with a median diameter of about 25 nm. The PEG layer was included in the nanoparticles' size estimation. From the corresponding TEM images, it can be also observed that the particles are predominantly spherical in shape (Figure 2A). Moreover, the high-resolution TEM image of a single PEG-reduced AgNP clearly displays the 5-nm PEG layer surrounding the atomically resolved silver core (Figure 2B).

### SERS measurements

**Figure 3:**
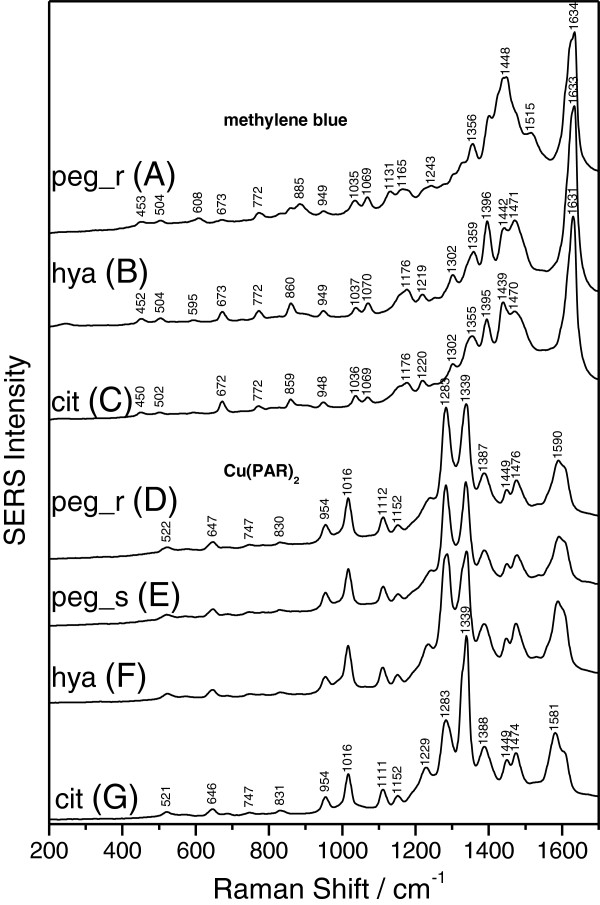
**SERS analysis of Cu(PAR)**_**2 **_**and methylene blue.** SERS spectra (employing the 532-nm laser line) of methylene blue adsorbed on (curve **A**) the rapid PEG-reduced (peg_r), (curve **B**) the hydroxylamine-reduced (hya), and (curve **C**) the citrate-reduced (cit) silver sol and of Cu(PAR)_2_ adsorbed on (curve **D**) the rapid PEG-reduced (peg_r), (curve **E**) the dropwise PEG-reduced (peg_s), (curve **F**) the hydroxylamine-reduced (hya), and (curve **G**) the citrate-reduced (cit) silver sol. The spectra were shifted for clarity. Specific vibrational peaks of analyte molecules are clearly visible for all three classes of silver colloids.

The SERS activity of the as-produced PEG-coated AgNPs is an important issue for further biomedical applications of these nanoparticles. Since both the citrate- and the hydroxylamine-reduced silver colloids are ones of the most used SERS substrates, they were chosen as a reference for the characterization of SERS activity of the PEG-reduced silver colloid. Figure 3 shows SERS spectra of methylene blue and Cu(PAR)_2_ analytes obtained with PEG-, citrate-, and hydroxylamine-reduced silver sols using the 532-nm laser line. The concentrations of methylene blue and Cu(PAR)_2_ analytes were 1.0 × 10^−6^ and 1.25 × 10^−5^ M, respectively. In order to achieve a higher SERS enhancement for citrate-reduced silver colloids, 10 μl of NaCl (0.1 M) solution was added. This was not the case for the PEG-reduced silver colloid, suggesting that the Raman signal is enhanced only by the single PEG-coated AgNP positioned in the laser focus and not by aggregates through the so-called *hot-spots*. The lack of pure Raman signal of the analytes, at the same concentrations as in the SERS spectra, supports the idea that the SERS signal is due to the presence of the PEG-coated nanoparticles.

**Figure 4:**
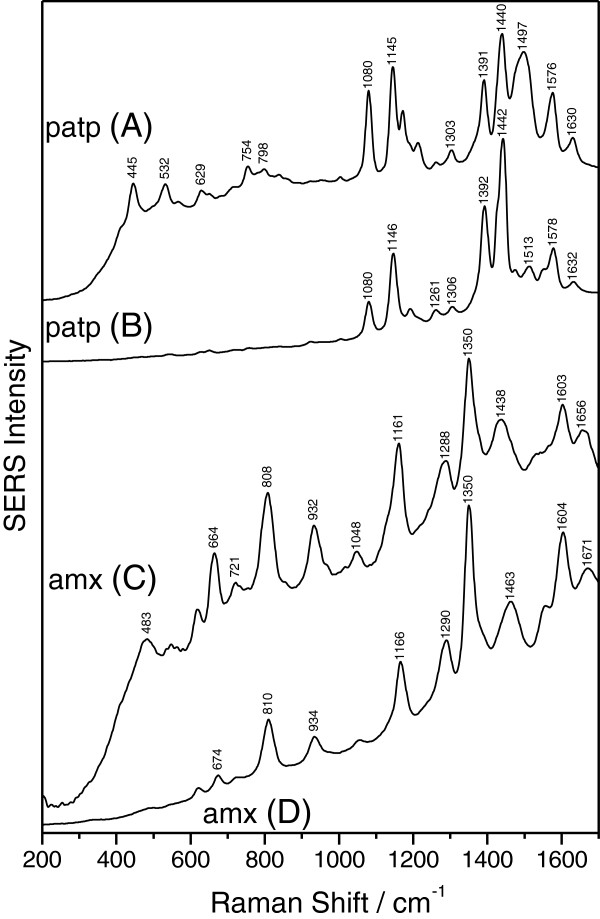
**SERS analysis of *p*-aminothiophenol and amoxicillin.** SERS spectra of *p*-aminothiophenol (patp) and amoxicillin (amx) adsorbed on PEG-reduced silver sol using both 633-nm (curves **A** and **C**) and 532-nm (curves **B** and **D**) laser lines. The spectra were shifted for clarity. Specific vibrational peaks of analytes molecules are clearly visible for all three classes of silver colloids.

The general applicability of the PEG-reduced silver sol is further checked by recording the SERS spectra of amoxicillin and *p*-aminothiophenol adsorbed on PEG-reduced silver sol, using both 532- and 633-nm laser lines (Figure 4). These spectra are then compared with those obtained on both the citrate- and the hydroxylamine-reduced silver colloid (Figure 4). The concentrations of amoxicillin and *p*-aminothiophenol analytes were 5 × 10^−5^ and 5 × 10^−7^ M, respectively.

The performed study reveals that the obtained SERS spectra yield similar shapes for the PEG-reduced silver colloid and both the citrate- and hydroxylamine-reduced silver colloids. For each analyte, the recorded peak position and the relative intensities in the recorded spectra were independent of the preparation method used to produce silver colloids. All investigated analytes adsorbed on the three classes of silver colloids gave comparable scattering intensities, indicating that the PEG-reduced silver colloid provides comparable SERS enhancement as conventional colloids.

## Conclusions

In this paper, we propose an easy, fast, one-step, facile, and green method for the synthesis of silver nanoparticles thus improving the straightforward creation of functionalized nanoparticles for biomedical usage. No toxic reagents, surfactant, and organic or inorganic solvents were implicated in the entire chemical trial. The successfully synthesized silver nanoparticles, which were produced using PEG 200 as reducing and stabilizing agents, own SERS-active properties. Though the procedure requires boiling conditions, the success of the experiment stands out throughout the speed in which biological clean nanoparticle systems can be synthesized in order to use them subsequently in analytical and biomedical applications. The major finding of this fast, one-step synthesis method resides in the use of additional -OH groups that are generated in the solution by sodium hydroxide, enhancing the speed of the chemical reduction of silver ions. The as-prepared PEG-coated silver nanoparticles showed a great stability in time.

## Abbreviations

AgNPs: Silver nanoparticles; PEG: Poly(ethylene glycol); SERS: Surface-enhanced Raman spectroscopy; TEM: Transmission electron microscopy.

## Competing interests

The authors declare that they have no competing interests.

## Authors’ contributions

RS and CML conceived and designed the experiments. GS, AGD, and CB carried out the synthesis of nanoparticles. GS and CI performed UV–vis spectroscopy and participated in SERS measurements. RS and NL performed TEM and SERS characterizations. RS, CI, CML, and NL drafted the manuscript. All authors read and approved the final manuscript.
